# Changes of Muscle-Irritability Dependent on the Blood Supply

**Published:** 1880-02

**Authors:** 


Changes of Muscle-Irritability Dependent on the Blood Supply.—In an article of somewhat ill-proportioned length, the following experimental results are given by Dr. J. Schmulewitch, (*Arch. f. Physiol.*, 1879, Heft 5 and 6, p. 479.)

Ligature of the aorta or crural artery increases slightly but positively the irritability of the muscles of the leg (rabbit) as tested with the induced current. The removal of the ligature and return of blood supply diminishes the irritability again below the standard of the normal leg. The author does of course not deny that the muscle ultimately dies if deprived of blood—his statements refer only to the immediate result. This influence of anæmia upon the muscle-irritability is manifested also, in case the motor nerve had previously been divided. It is hence not due to any action of the spinal cord.

A transitory increase of the direct muscle irritability is also caused by section of the motor nerve. This effect is due also to anæmia caused by the temporary state of excitation of the vaso-constrictor fibres contained in the motor trunk. Hence no further rise of irritability of the nerve section occurs if the aorta is left ligated during the experiment. This view is further corroborated by the fact that section of the motor nerve is of effect upon the irritability of the muscle, even though the animal be curarized, provided the dose is too small to paralyze the vaso-motor fibres.—*Journal of Nervous and Mental Disease.*

